# Erythrocyte Enrichment in Hematopoietic Progenitor Cell Cultures Based on Magnetic Susceptibility of the Hemoglobin

**DOI:** 10.1371/journal.pone.0039491

**Published:** 2012-08-27

**Authors:** Xiaoxia Jin, Stewart Abbot, Xiaokui Zhang, Lin Kang, Vanessa Voskinarian-Berse, Rui Zhao, Marina V. Kameneva, Lee R. Moore, Jeffrey J. Chalmers, Maciej Zborowski

**Affiliations:** 1 Department of Biomedical Engineering, Cleveland Clinic, Cleveland, Ohio, United States of America; 2 William G. Lowrie Department of Chemical and Biomolecular Engineering, The Ohio State University, Columbus, Ohio, United States of America; 3 Celgene Cellular Therapeutics, Warren, New Jersey, United States of America; 4 McGowan Institute for Regenerative Medicine, University of Pittsburgh, Pittsburgh, Pennsylvania, United States of America; Tufts University, United States of America

## Abstract

Using novel media formulations, it has been demonstrated that human placenta and umbilical cord blood-derived CD34+ cells can be expanded and differentiated into erythroid cells with high efficiency. However, obtaining mature and functional erythrocytes from the immature cell cultures with high purity and in an efficient manner remains a significant challenge. A distinguishing feature of a reticulocyte and maturing erythrocyte is the increasing concentration of hemoglobin and decreasing cell volume that results in increased cell magnetophoretic mobility (MM) when exposed to high magnetic fields and gradients, under anoxic conditions. Taking advantage of these initial observations, we studied a noninvasive (label-free) magnetic separation and analysis process to enrich and identify cultured functional erythrocytes. In addition to the magnetic cell separation and cell motion analysis in the magnetic field, the cell cultures were characterized for cell sedimentation rate, cell volume distributions using differential interference microscopy, immunophenotyping (glycophorin A), hemoglobin concentration and shear-induced deformability (elongation index, EI, by ektacytometry) to test for mature erythrocyte attributes. A commercial, packed column high-gradient magnetic separator (HGMS) was used for magnetic separation. The magnetically enriched fraction comprised 80% of the maturing cells (predominantly reticulocytes) that showed near 70% overlap of EI with the reference cord blood-derived RBC and over 50% overlap with the adult donor RBCs. The results demonstrate feasibility of label-free magnetic enrichment of erythrocyte fraction of CD34+ progenitor-derived cultures based on the presence of paramagnetic hemoglobin in the maturing erythrocytes.

## Introduction

Red blood cells (RBCs) make up 40 to 50 percent of the average human blood volume and are the most commonly transfused blood product, with 40,000 RBC units (∼220 mL) used in the United States every day [1]. The difficulty in meeting the high demand is related to the limited supply of the RBCs, lack of availability of certain phenotypes and the possibility of infection, which continue to create interest in RBC susbstitutes and alternative sources of RBCs for transfusion. A number of recent studies have suggested the possibility of *ex vivo* erythrogenesis from hematopoietic stem cells (HSCs) isolated from peripheral blood, bone marrow, and umbilical cord collected following delivery [2], [3]. This approach could provide the basis for large-scale RBC production, in combination with a suitable protocol for HSC expansion and staged erythrocytic differentiation. HSCs are typically identified by a cluster of differentiation 34 (CD34) surface marker and can be isolated immunomagnetically from cord blood and placenta derived cell populations, then cultivated using novel culture media formulations in standard culture systems or bioreactors that can mimic bone marrow microenvironment [4]. After substantial expansion, HSCs can be induced to differentiate into mature, functional RBCs. Given the complexity of mammalian erythropoiesis, it is difficult to constrain cultured HSCs to commit exclusively to the erythroid line and homogeneously differentiate and mature into enucleated RBC populations. Thus, obtaining mature and functional erythrocytes from cultured HSC populations with high purity remains a challenge.

Large scale RBC production in culture requires continuous removal of the maturing erythroid cells from the cell culture mixture. The conventional adult RBC separation methods based on differential cell sedimentation rate (by centrifugation) or size (by counter-current elutriation) do not work efficiently on maturing erythroid cells because their physical characteristics is not sufficiently differentiated from those of the progenitor cells (Figure S1). The known differences between the immunophenotype of the differentiated erythroid cell and the progenitor cells lend themselves to fluorescence-activated cell sorting (FACS) or immunomagnetic nanoparticle tagging and magnetic separation, however, considering the sheer cell volume required for large scale RBC production and the cost limitations such methods may be too lengthy, too costly and potentially prone to contamination because of large volume of the labeling reagents required for practical applications. In this study, we have tested feasibility of using paramagnetic property of deoxygenated hemoglobin as a distinguishing feature of maturing erythroid cells in the mixture of diamagnetic, early progenitor cells (as illustrated in Figure S1) for label-free, magnetic separation. As early as 1936 Pauling and coworkers described that deoxygenated hemoglobin and methemoglobin (metHb) are paramagnetic [5] because of the presence of unpaired electrons in the four heme groups. In contrast, due to its covalent bonds, oxygenated hemoglobin (oxy Hb) has no unpaired electrons and is diamagnetic. High gradient magnetic separators were used in the past to demonstrate feasibility of adult RBC enrichment from whole blood deoxygenated by nitrogen gas [6], [7], [8], [9], [10].

The magnetic composition of the cell culture mixture and the enrichment of cells containing functional hemoglobin was determined by measuring cell magnetophoretic mobility (MM) distribution in the cell sample before and after separation. MM analyses were performed using cell tracking velocimetry (CTV), an analytical instrument developed in the course of previous studies that is capable of generating MM histograms and 2-D plots of cell MM against cell sedimentation rate (velocity) [11]. Other physical RBC attributes that distinguish them from HSC and erythroblasts, such as size, morphology, immunophenotype, and deformability, were also assessed and compared before and after magnetic separation.

## Materials and Methods

### Magnetophoretic mobility

Magnetophoretic mobility, *m*, (also denoted by MM in the text) is defined as a ratio of the field-induced velocity, *u_m_*, and the local magnetostatic energy density gradient, *S_m_*:
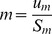
(1)where *S_m_* = 146 ± 1 T.A/mm^2^ is nearly constant in the region of interest (1.05 mm wide×0.79 mm high). For paramagnetic and diamagnetic cells, Eq. 1 reduces to an expression that depends only on cell and the suspending fluid properties:
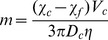
(2)where *η* = 0.93×10^−3^ kg/m-s is the aqueous solution viscosity, *V_c_* and *D_c_* are cell volume and hydrodynamic diameter, *χ_c_* and *χ_f_* are volume magnetic susceptibility of a cell and media, respectively. Here *χ_f_*  = −9.04×10^−6^ (SI). Notably, *χ_oxyRBC_−χ_f_*<0 for the fully oxygenated RBC, and *χ_deoxyRBC_−χ_f_* >0, *χ_metHbRBC_−χ_f_* >0 for the fully deoxygenated and methemoglobinated RBC, respectively (see File S1). Consequently, we expect that the oxy RBCs in solution are pushed away by the magnet (with negative MM value), and the deoxy RBCs and metHb RBCs are attracted by the magnet (with positive MM value).

### Settling velocity

Considering a simple model of spherical cell falling through a motionless fluid, its immersed weight can be balanced by the drag force which is given by the Stokes equation:
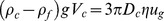
(3)Here *D_c_* is interpreted as the “equivalent hydrodynamic diameter” of the RBC that results from averaging of a large number of RBC sedimentation velocity measurements to account for the complex shape of the RBC (a biconcave disc). The particle sedimentation coefficient, *s*, is defined as
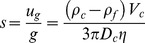
(4)where *u_g_* is the settling velocity, *g* = 9.81 m/s^2^ is the standard gravitational acceleration, *ρ_c_* and *ρ_f_* are densities of the cell and suspending medium.

Thus, the diameter of a sedimenting cell can be calculated by measuring cell settling velocity, or its sedimentation coefficient:
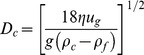
(5)


**Figure 1:**
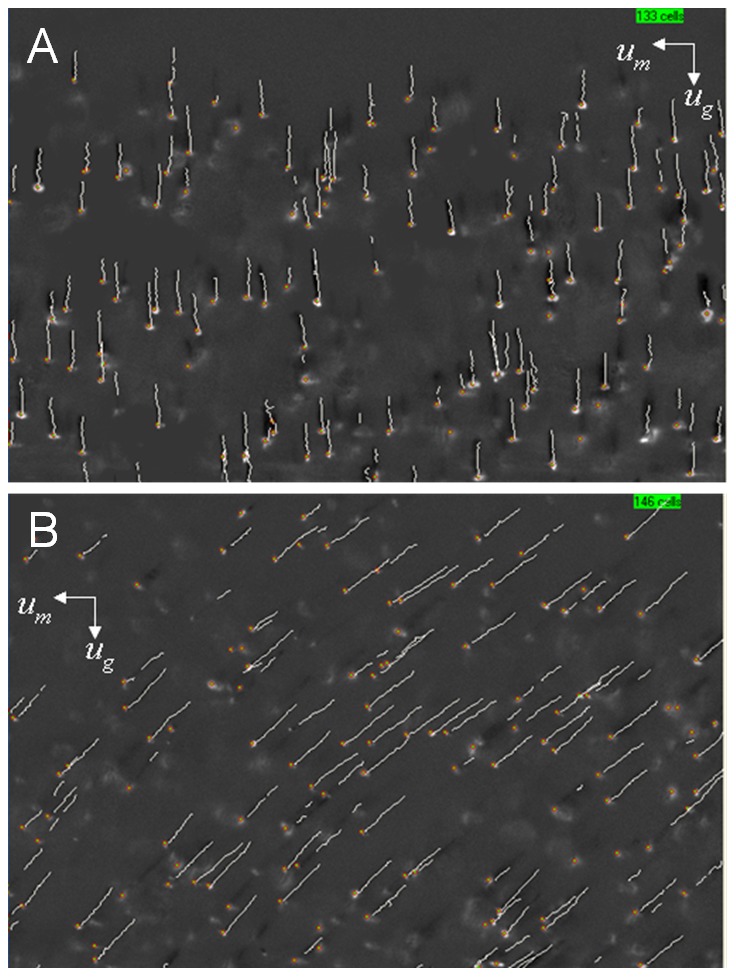
Strong magnetic field gradient deflects sedimentation trajectories of high spin hemoglobin RBCs. Examples of the computer screen output of the CTV software showing the trajectories of A) oxygenated RBCs (low spin hemoglobin) and B) methemoglobin-conatining RBCs (high spin hemoglobin). Note the horizontal component of the cell trajectory due to cell magnetophoresis along the horizontal lines of the magnetic force. The cell magnetophoretic mobility, *m*, was defined as the horizontal distance traveled by the cell divided by the time of the image sequence acquisition and by the local magnetic energy density gradient (see Eqn 1 in text).

**Figure 2:**
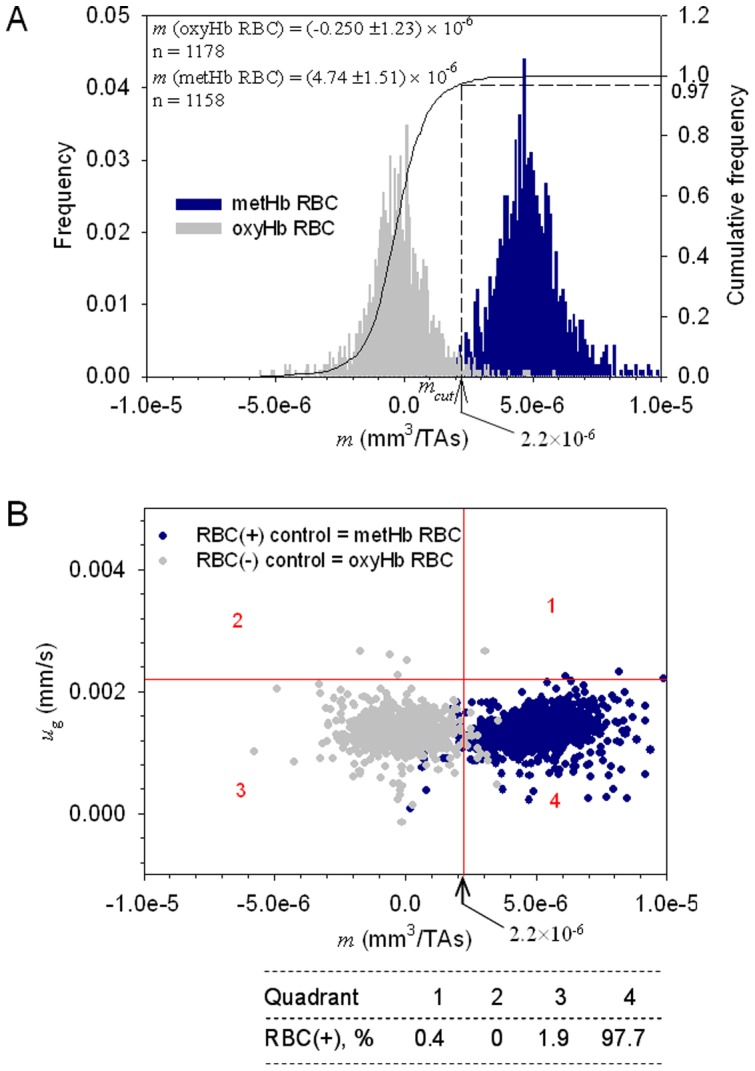
Donor blood RBC magnetophoresis as a control for the hematopoietic stem cell (HSC) culture analysis. MM histograms (A) and dot plots of MM vs. cell sedimentation velocity (B) of the oxygenated and the metHb containing donor RBCs used as negative and positive controls, respectively, for the cell magnetophoretic mobility analysis. The cut-off mobility, mcut = 2.2×10−6 mm3/T.A.s, that separates the magnetic from non-magnetic fractions was determined from the cumulative frequency distribution of oxyHb RBC at 0.97 value.

CTV is capable of measuring *m* and *u*_g_ (or *s*) simultaneously for hundreds to thousands cells on a cell by cell basis, which allows us to differentiate small, magnetic RBCs or RBC-like cells from bigger, diamagnetic non-RBC cells or undifferentiated, erythroid cells (compare Figures 1, 2 and Figure S1). The 2-D dot plots of cell *u_g_* versus cell *m* and the associated quadrant statistics were generated to calculate differences in fractional composition of small, magnetically susceptible cells (putative mature RBCs and reticulocytes) and large, non-susceptible cells (putative progenitor cells with no functional hemoglobin) between the unsorted and magnetically sorted fractions (Figure 2).

### Estimation of intracellular Hb concentration in cultured RBCs

The parameter *m* of a deoxy or metHb RBC is directly proportional to cell magnetic susceptibility (Eqn (2)), which is a linear function of the cell deoxy Hb or metHb content (File S1 and Eqns (1) or (3) therein). When a deoxy Hb or metHb containing RBC is placed in the CTV system, where the magnetic force is orthogonal to gravity, the RBC moves in the horizontal direction caused by magnetic energy density gradient, Eqn (1), as well as vertical direction induced by gravitational acceleration, Eqn (3), as illustrated in Figure 1. Dividing Eqn (4) by Eqn (2), one eliminates*η*, *D_c_*, and *V*_c_ to obtain:
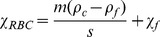
(6)Subsequently, combining File S1 Eqns (1) or (3) and (2) therein with the above Eqn (6), one obtains expression for Hb concentration for a single cultured RBC if density of RBC and physical properties of medium are known:
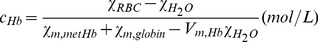
(7)With the molecular weight of hemoglobin (*M_w,Hb_* = 64,450 g/mol) and the measured cell magnetic susceptibility and volume data, one can obtain a value for mean corpuscular hemoglobin (MCH, pg/RBC), which is the average mass of hemoglobin per RBC, or mean corpuscular hemoglobin concentration (MCHC, g/dL RBC), which is the concentration of hemoglobin in a given volume of packed RBC. In this manner, the RBC magnetophoresis measurement by CTV is capable of providing information on hemoglobin concentration in maturing, cultured RBC that is accumulated gradually during erythropoiesis.

### Cell sources

#### Ethics statement

Postpartum placentas were procured by Celgene Cellular Therepeutics (CCT) under written informed consent, including donor eligibility documentation. CCT's procurement program for the acquisition of placental tissue has been reviewed by a leading NY area hospital's IRB (Saint Barnabus Hospital). The blood samples underwent a series of quality control tests, including serology, bacteriology and HLA typing (results not shown). Total nucleated cells as the source of HSCs were generated by treating the donor matched umbilical cord blood and placenta derived stem cells with ammonium chloride (catalog# 07850, StemCell Technologies). The subsequent purification of HSCs was performed using RoboSep® automated cell isolation system and EasySep® Human Progenitor Cell Enrichment Kit (catalog# 19056, StemCell Technologies). Expansion and differentiation of HSCs into RBCs were performed as previously described [3], [12]. The cultures were shipped by surface courier overnight to the laboratory of M.Z. Once received, HSC-derived RBC cultures were centrifuged once at 250 g for 10 minutes. The cell pellet was then suspended in Ca^2+^, Mg^2+^-free Dulbecco's phosphate-buffered saline (PBS) containing 0.5% Bovine Serum Albumin (BSA, Sigma, St. Louis, MO) and 2 mM Ethylene Diamine Tetraacetic Acid (EDTA, Sigma, St. Louis, MO). The cell suspension was filtered by a 40 µm cell strain (BD Biosciences, Durham, NC) to remove cell aggregates. The cell concentration was determined and a desired concentration for separation was prepared.

The whole blood (WB) was received from Cleveland Clinic Blood Banking and Transfusion Medicine under an Institutional Review Board (IRB) approved protocol for blood collection from normal volunteers for research. A stock suspension was prepared by diluting 0.1 mL whole blood with 10 mL PBS. An aliquot of 2.0×10^6^ RBCs from the stock suspension was used as the donor-derived oxy RBC control (negative control).

### Irreversible RBC magnetization

A 5 mM oxidant solution was prepared by dissolving sodium nitrite (NaNO_2_, Sigma-Aldrich Co., Milwaukee, WI) in PBS at room temperature. An aliquot of 2.5×10^6^ RBCs from the RBC stock suspension prepared as described above was pelleted and resuspended in 10 mL of 5 mM sodium nitrite solution, which was then incubated for about 1.5 hours to achieve a 100% methemoglobin oxidation. An aliquot of HSC-derived RBC cultures with same cell number was oxidatively treated at the same way: suspended in 10 mL of 5 mM NaNO_2_ solution, 1.5 hours. After incubation, methemoglobinated RBCs or HSC-derived RBC cultures were washed once and resuspended in 5 mL PBS containing 0.1% Pluronic F-68 (Sigma-Aldrich, St Louis, MO) for CTV analysis. Methemoglobinated donor-derived RBC was regarded as positive control.

### Reversible RBC magnetization by deoxygenation of HSC-derived RBC cultures

A Glove-Bag™ inflatable glove chamber (Cole Parmer, Vernon Hills, IL), filled with nitrogen (Medipure™ nitrogen, concentration >99%, Praxair, Inc., Danbury, CT) was used to deoxygenate HSC- derived RBC cultures (Figure S2). Before deoxygenation, all materials and equipment including the separation system, degassed sterile buffer (PBS +2 mM EDTA +0.5% BSA), and sterile collection tubes were placed in the glove bag, which was then tightly sealed. Additional details are provided in File S1.

### Magnetic cell separation

A commercial magnetic separation system (QuadroMACS™ Separator combining four MidiMACS™ separation units and LD columns, Miltenyi Biotec, Auburn, CA) was used for magnetic RBC enrichment from HSC-derived RBC cultures. Deoxygenated cultures were loaded directly into a MACS® LD column which was placed in the QuadroMACS™ separator kept under anoxic conditions inside the inflatable glove chamber filled with N_2_ gas (Figure S2). Cells which passed through the column contained within the magnet are labeled as negative fraction and they are expected to be “non-magnetic”, including HSCs and erythroid cells before final maturation. The cells retained in the separation column are labeled as positive fraction, which is “magnetic” and expected to consist of maturing RBC-like cells nearly full of functional hemoglobin. They were eluted from LD column after its removal from the magnet. Once separation was finished, oxygenated cells were reversibly recovered by exposing the collected cells to air.

### CTV analysis

The CTV instrument previously developed and tested [13], [14], [15] was used to measure magnetophoretic mobility and settling velocity of the donor-derived RBC controls, oxy RBC and metHb RBC. The cell number concentration was kept at 0.4×10^6^ cells/mL. The unsorted and sorted HSC- derived RBC cultures were exposed to oxidative treatment to convert hemoglobin to methemoglobin in order to identify the hemoglobin-containing cells (the putative maturing RBCs) by CTV analysis, and to evaluate the separation performance. More than 1,000 cells in each sample were analyzed.

### Cell concentration and size distribution

An automated cell counter, Z2™ Coulter Counter® (Beckman Coulter Inc., Fullerton, CA, USA), with a 70 µm aperture, a sample volume of 0.5 mL and a diameter range setting between 4 and 14 µm was used to measure cell concentration and size distribution.

### Cell morphology analysis by differential interference contrast (DIC) microscopy

Ten µL samples were placed on slides and covered by covers slip, which were then visualized by a Leica DMR upright microscope equipped with a 40× objective lens and a CCD camera provided by the Imaging Core of Lerner Research Institute in Cleveland Clinic. The software, ImagePro Plus was used to acquire and process the DIC images. Unsorted and sorted samples were analyzed and compared. The RBC images from donor blood were used as a control.

### RBC deformability

An important step toward the large-scale application of the cultured RBCs is characterization of their transport function, especially their ability to enter and pass smallest capillaries which depends on RBC deformability. Mature RBCs have the ability to deform under the influence of externally applied shear stresses. RBC deformability was directly assessed using Linkam CSS450 Optical Shearing System (Linkam Scientific Instruments Ltd, United Kingdom) [16].

Positively and negatively separated cells were suspended in a polyvinylpyrrolidone (PVP) solution (∼10^6^ cells in 80 µL) and loaded in the sample unit of the Linkam CSS450 shearing stage. The shearing stage was mounted to a microscope (Olympus BH2 upright microscope) sub-stage. A long distance objective with a 40× magnification was used to visualize the cells. Linkam operation was controlled by an IBM PC generating shear rates from 10 to 1500 s^−1^. A PerkinElmer X400 stroboscope (PerkinElmer Corp., Waltham, MA, USA) was used as illuminator and a PCO CCD camera (PCO Corp, Kelheim, Germany) was attached to the microscope to capture the images and send them to PC. Both the stroboscope and the camera were synchronized by a pulse generator. An NIH software ImageJ was employed to analyze the images and obtain cell deformation and orientation data.

## Results

### Deoxygenation of whole blood

Deoxygenation of 5 mL whole blood in one 50 mL rotating conical tube was tested in the N_2_ filled glove bag. After 3 hr exposure to N_2_, blood oxygen partial pressure (pO_2_) dropped to 2.3 mmHg (the pO_2_ in room air is approximately 156 mmHg), blood oxygen saturation (sO_2_) dropped to 3.9% (from the initial value of 99%), as shown in Figure S3. The mean RBC number concentration is 5×10^9^/mL of whole blood [17], which is much higher than that expected in HSC-derived RBC cultures; consequently, it was assumed that this method would deoxygenate the cultures equally well or better. Five mL of HSC-derived RBC cultures with a cell number concentration of (1–2)×10^8^/mL were placed in the same type of 50 mL, inclined, rotating conical tube and exposed to N_2_ atmosphere for 3 hr, following which all mature RBCs in HSC-derived RBC culture were considered to be completely deoxygenated. After deoxygenation, cultures were isolated by MACS separation in the glove bag (File S1 and Figure S2).

### Differences in MM between oxyHb RBC and metHb RBC from donor blood

**Figure 3:**
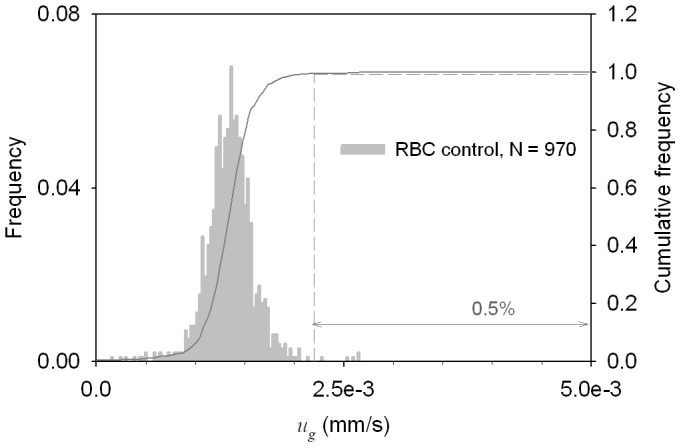
Donor blood oxyHb RBC settling velocity as a control for HSC culture analysis. The blood settling velocity histogram and its cumulative frequency distribution are shown. The cut-off settling velocity was set at 0.995 cumulative frequency for the subsequent HSC culture analysis.

As presented previously, the magnetic susceptibility of erythrocyte is related to the hemoglobin magnetic susceptibility which, in turn, depends on oxygen binding to the heme group. An oxygenated RBC is more diamagnetic than the suspending, aqueous physiologic electrolyte solution while a deoxygenated or metHb-containing RBC is less diamagnetic due to paramagnetic contribution of the metHb (File S1 and Eqns (4)–(6) therein) resulting in a negative MM of the oxyHb RBC and a positive MM of the metHb RBC (Eqn (2) above). These theoretical predictions were experimentally validated using CTV measurements of oxygenated and metHb suspensions of donor RBCs (obtained from discarded blood from patients treated by therapeutic phlebotomy, with the IRB approval). Figure 2A presents histograms of the CTV determined MM of metHb containing RBC and oxygenated RBC (equilibrated with air). As expected, a clear difference between the populations can be observed. The same data are replotted in Figure 2B as a scatterplot, with the addition of the vertical axis showing cell sedimentation velocity, *u_g_*. Again, the two data sets (metHb and oxyHb RBCs) are clearly discriminated. The likely source of the data dispersion is the heterogeneous nature of the normal RBC population and the background noise introduced by CTV. Therefore, the cumulative frequency plot for oxyHb RBC distribution, Figure 2A, was used for the determination of a cut-off MM value, to discriminate between the oxyHb RBCs and metHb RBCs. Here, the cut-off MM was assumed as corresponding to 97% of the oxyHb RBC cumulative frequency, *m_cut_* = 2.2×10^−6^ mm^3^/T.A.s, Figure 2A. In other words, less than 3% of oxyHb RBCs are expected to be magnetically mobile, consistent with the low metHb RBC fraction known to be present in donor blood. Subsequently, the cut-off MM was used to divide the scatterplot shown in Figure 2B into quadrants, and to calculate the quadrant statistics (Figure 2B). The cut-off sedimentation velocity value, 0.0022 mm/s, used to draw the horizontal line of the quadrants in Figure 2B, was likewise selected based on the statistics of the sedimentation velocity distribution, and was set at 99.5% cumulative frequency of that distribution, Figure 3. This is consistent with the expected nearly 100% cell population viability in the donor RBCs.

This experimental, statistical, and theoretical analysis of the cell motion in the CTV apparatus allows us to interpret the cell MM and sedimentation velocity data in terms of the cell physical properties: its functional Hb content and size, characteristic of the cell differentiation stage (Figure S1). According to this interpretation, Quadrant 1 in Figure 2B comprises large cells with functional Hb (putative reticulocytes and some normoblasts); Quadrant 2 comprises large cells with no functional Hb (progenitor cells); Quadrant 3 comprises small cells with no functional Hb (putative cell ghosts, cells that do not belong to the erythroid lineage, expelled nuclei and other artifacts of the cell culture), and Quandrant 4 comprises mature RBCs.

The type of analysis illustrated in Figure 2 allows us to establish reference quandrants based on cut-off values for negative control (oxyHb RBCs) and a positive control (metHb RBCs) for normal donor blood. The establishment of these quandrants subsequently allows us to functionally characterize RBC produced from cultures of hematopoetic stem cells and make predictions about cellular composition of the magnetically separated fractions. It is worth noting here the high sensitivity of the CTV instrument, sufficient to detect changes in the individual cell motion caused by changes in the hemoglobin iron spin state. This allowed us to develop type of quadrant statistics for cell data interpretation similar to that used routinely in flow cytometry [18].

### CTV as a tool for determination of RBC-like cells in HSC cultures

**Figure 4:**
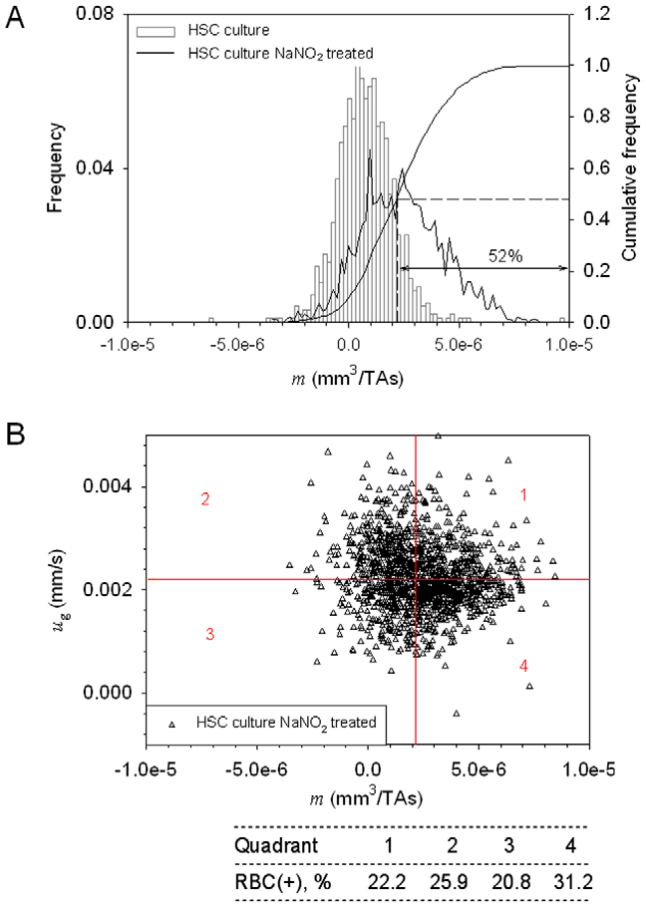
Conversion to methemoglobin by oxidative treatment of HSC culture increases the magnetic cell fractional concentration. MM histograms (A) and dot plots (B) of HSC culture before and after the oxidative treatment used for conversion of Hb to paramagnetic metHb and used to determine the fractional concentration of maturing RBCs in culture.

Prior to separation of HSC-derived RBC cultures in deoxygenated condition, the presence of the hemoglobin-containing cells was established by oxidative treatment with NaNO_2_ of the cell sample aliquot to convert the hemoglobin to methemoglobin. These cell preparations were then analyzed by CTV for MM and sedimentation rate. Figure 4A shows the MM histograms of the cell culture before and after oxidative treatment. Note shift in the mobility distribution to the right, as expected, of a sample preparation with cells that contain metHb. Compared with the normal donor RBC negative (oxyHb RBC) and positive (metHb RBC) controls (Figure 2), the MM distribution of the cell from the oxidatively treated HSC- derived RBC cultures suggests the presence of cells with a whole spectrum of hemoglobin concentration, from none to a maximum concentration characteristic of mature RBCs. Compared with Figure S1, the MM distribution indicated significant presence of cells with hemoglobin content equivalent to mature RBCs, reticulocytes and normoblasts in the HSC-derived RBC cultures. An estimate of the percent of cells containing functional hemoglobin was determined from the cumulative frequency plot in Figure 2A; at a *m_cut_* = 2.2×10^−6^ mm^3^/T.A.s, resulting in the value of 52%.

Replotting the data as a scatterplot by the addition of the cell sedimentation velocity axis, Figure 4B, revealed that a significant fraction of cells containing the functional hemoglobin are larger than the mature RBCs, compare Quadrant 1 in Figure 4B with Quandrant 1 in Figure 2B. Thus from Figure 4B, the cell culture sample comprised 22.2% reticulocytes (Quadrant 1), 25.9% progenitor cells (Quadrant 2), 20.8% unidentified cells (Quadrant 3) and 31.2% putative, mature RBCs (Quadrant 4).

### CTV analysis of magnetically fractioned, deoxygented HSC cultures in MACS LD columns

**Figure 5:**
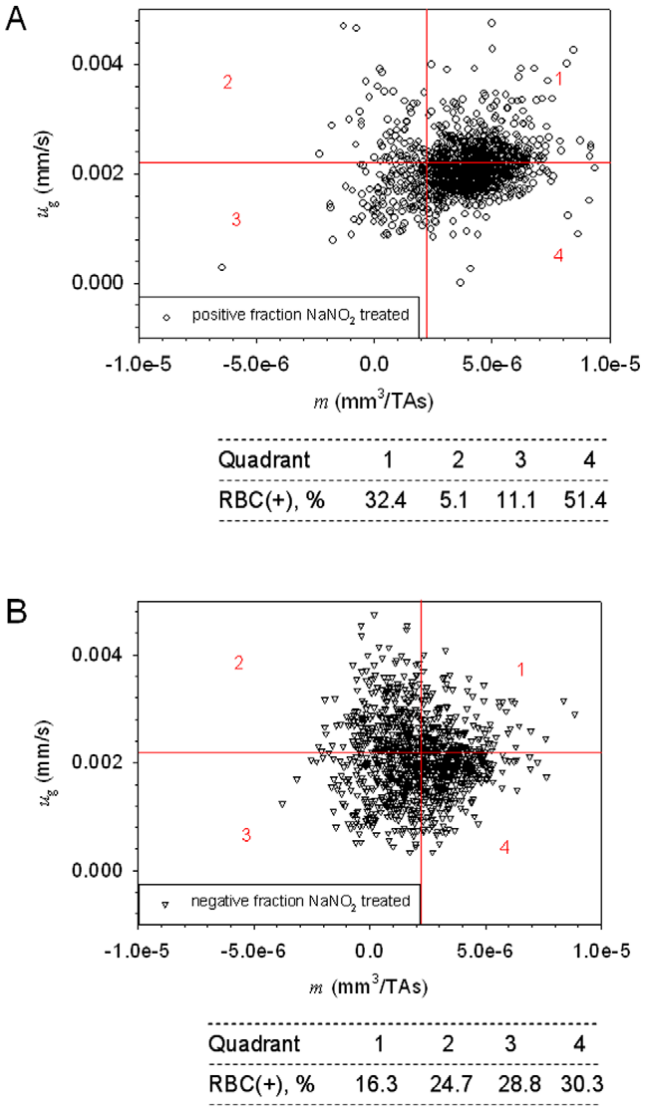
Magnetic separation of HSC cultures increases concentration of the hemoglobin containing cells. Dot plots of sorted HSC culture under anoxic conditions (in N2 atmosphere). (A) Positive fraction, (B) negative fraction showing shift towards more magnetic, smaller cells (putative maturing RBCs) in the “positive” fraction.

The same CTV analysis was repeated on cell fractions collected from the magnetic column after magnetic separation of the HSC cultures using a MACS LD column. Representative examples of dot plots of sorted fractions are shown in Figure 5A and 5B. Most cells retained in the column (the “positive” fraction) are magnetic, Figure 5A, as suggested by the shift of the data distribution towards the right when compared with the original sample, Figure 4B. The dot plot statistics of Figure 5A shows an increase in percentage of cells in Quadrants 1+4 as compared to the original sample, from 53.4% to 83.8% suggesting enrichment of the hemoglobin containing cells in the magnetically retained (“positive”) cell fraction. When limited to changes in Quadrant 4 (representative of the mature RBCs only) the increase was from 31.2% to 51.4% indicating enrichment of the mature RBCs. Notable is significant depletion of large cells without hemoglobin in the magnetically retained fraction, compare Quadrants 2 in Figures 4A and 5A, from 25.9% to 5.1%, as would be expected. Rather unexpected, however, were losses of the hemoglobin-containing cells in the unretained (“negative”) fraction, seen in Quadrants 1 and 4 in Figure 5B. This suggests that the magnetic column was effective in retaining high magnetic susceptibility cells (contributing to the right-hand tail of the magnetophoretic mobility distribution shown in Figure 4A) but less so in capturing the less susceptible ones (whose mobility is only moderately higher than *m_cut_* = 2.2×10^−6^ mm^3^/T.A.s in Figure 4A). Considering that the cell MM frequency distribution decreases monotonically for MM>*m_cut_*, Figure 4A, one indeed may expect to see significant losses of weakly magnetic cells in the non-retained fraction, as borne out by the experiment.

Qualitatively, the dot plot of positive fraction, Figure 5A, has a similar distribution as that of the RBC positive control, Figure 2B, Quadrant 4, except of the shift towards higher settling velocity values. This indicates that the magnetically isolated cells contain similar amount of hemoglobin as the donor-derived RBCs, but have bigger size. This observation was further confirmed by the Coulter counter and morphological analyses, described below. In contrast, cells in the negative fraction are on average less magnetic and are larger as compared to the positive fraction, Figure 5B.

### Cell size analysis by Coulter counter and CTV

**Figure 6:**
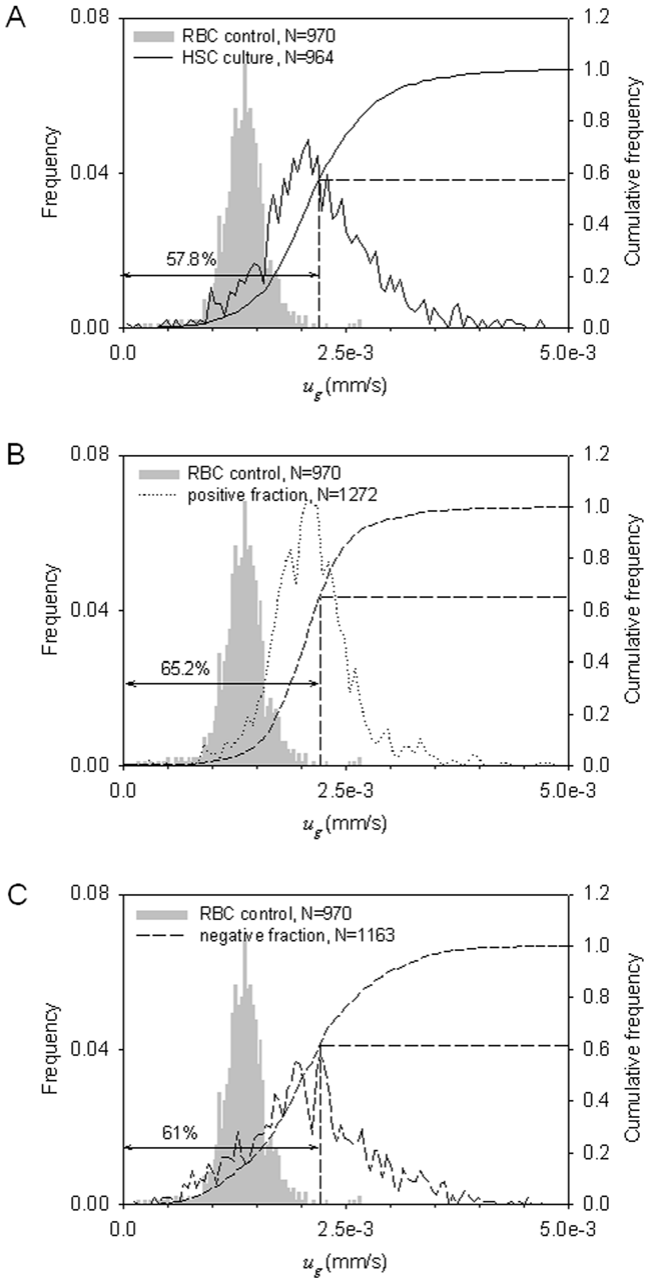
Magnetic separation of HSC cultures increases concentration of fast sedimenting cells. Cell volume distributions by settling velocity measurements with CTV: the unsorted cells (A), “positive” cell fraction (B), and the “negative” cell fraction (C) against the donor RBC control. The comparison shows small increase in the fast sedimenting cell fraction in the “positive” fraction, in agreement with the Coulter counter results shown in the Figure S4.

Cell size in the unsorted and sorted samples were evaluated by Coulter counter and CTV. Figure S4 presents the cell volume distribution of the HSC-derived RBC culture before and after MACS separation. Notable is larger mean size of cells as compared to the donor-derived RBC control (marked in grey in Figure S4A) and a broader cell size distribution. The narrower distribution of the positive fraction as compared to that of the negative fraction, Figure S4B, suggests a more homogenous cell composition in the positive fraction, consistent with the MM results, discussed above. Also notable is the large mean cellular volume (MCV, ≈ 200 fL) of the positive fraction that is much bigger than that of normal donor-derived RBCs, reported in the literature, which is in the range of 80–100 fL [17]. This agrees with the shift to the right of the positive cell fraction after magnetic separation of the HSC-derived RBC culture, relative to the positive RBC control, discussed above in reference to Figure S4A. While appreciating that the settling velocity is directly proportional to cell volume (Eqn (4)), the phenomenon is further confirmed by analyzing shifts in the settling velocity distributions measured by CTV shown in Figure 6. Unsorted cells (Figure 6 A) and magnetically unretained cells (“negative” fraction, Figure 6C) have a broader settling velocity distribution. The selected positive cells (Figure 6B) sedimented a little faster in the gravity field than the normal donor-derived RBCs, however. This again indicates that the enriched cells are bigger than the donor-derived RBCs.

### Hemoglobin content in enriched RBC-like cells

The data for hemoglobin containing cells, selected by the condition that their MM>*m_cut_* = 2.2×10^−6^ mm^3^/T.A.s (Quadrants 1 and 4 in Figure 5A) were used for the intracellular Hb content calculation, using Eqn (7) as discussed above.

**Figure 7:**
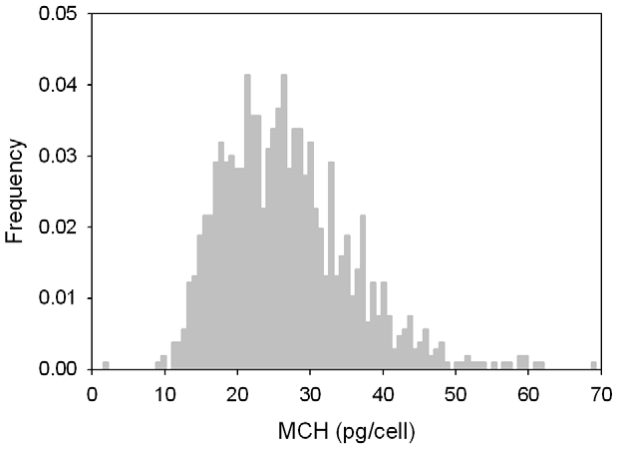
MCHC distribution of the magnetically enriched cells falls within the range expected of the maturing RBCs (compare with Figure S1).

The mass density of these Hb containing cells was determined by applying the mean cell diameter (7.26 µm) from the Coulter counter analysis and the mean settling velocity (2.12×10^−3^ mm/s) from CTV analysis to Eqn (4). A density of 1.070 g/cm^3^, somewhat smaller than that of mature RBCs, was obtained, which was then used to calculate the diameter and volume of Hb containing cells from the settling velocity data. With this value of density, and Eqn (6) and (7), the corpuscular hemoglobin concentration was calculated for each cell in Quadrant 1 and 4 (Figure 5A). The corresponding corpuscular hemoglobin histogram is presented in Figure 7. A mean corpuscular hemoglobin (MCH) was determined to be 26.6 pg/cell, with a standard deviation of 9.0 pg/cell. These values are very close to the published MCH values of normal donor-derived RBC, which are in a range of 27.5–33.2 pg/cell [17]. This confirms consistency of the cell MM data and our CTV analysis with the published data on intracellular iron in the mature RBCs.

### Morphology and deformability analysis

**Figure 8:**
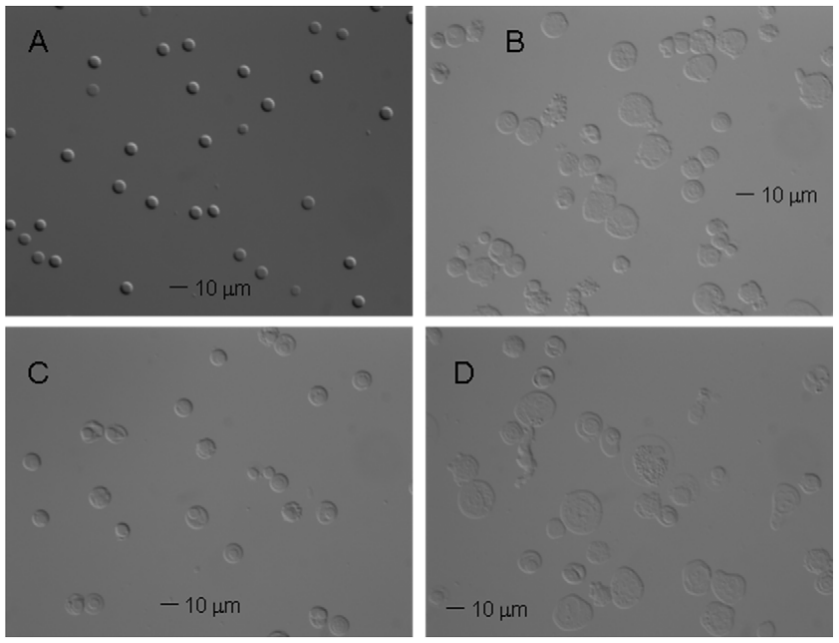
Magnetic separation of HSC cultures results in the improved morphology of the cells. The morphology of cells by differential interefence contrast (DIC) microscopy. (A) The donor-derived RBCs as a control and the HSC culture before (B) and after separation: (C) “positive” fraction; (D) “negative” fraction. Note enrichment in smaller, denser cells in (C) consistent with the presence of maturing RBCs in the magnetically separated “positive” fraction. (Their size and lack of evidence of the biconcave disk morphology suggests that these are reticulocytes rather than fully mature RBCs.).

DIC microscopy is an optical microscopy illumination technique used to enhance the contrast in unstained, transparent cell samples in their natural milieu (aqueous solutions). The image has a very realistic, clear, three-dimensional appearance almost entirely free of the phase contrast artifacts of phase halo and shading compared to a phase contrast image [19]. Figure 8 presents examples of DIC images of unsorted and sorted cell samples. One can note that the original cell population of HSC-derived RBC culture (panel B) is heterogeneous, with a very small fraction of RBC-like cells, as compared to the blood donor RBC control (panel A). In contrast, the morphology of the magnetically separated positive cell fraction (panel C) is very homogeneous, with increased frequency of the small, round, RBC like cells as compared to the original sample and the magnetic negative fraction (panel D). These results confirm the results of CTV and Coulter counter analysis, presented above.

**Figure 9:**
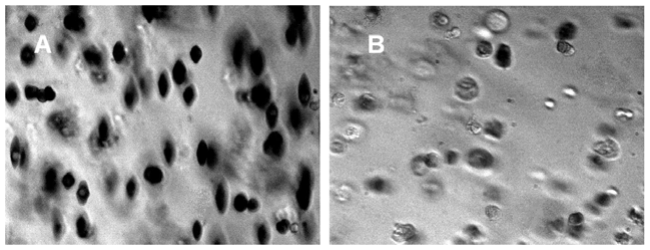
Magnetic separation of HSC cultures improves viscoelastic properties of the cells. Deformability of cells in positive fraction (A) and negative fraction (B). Note increased frequency of dark, elongated objects, associated with hemoglobin containing deformable cells characteristic of mature RBC, in panel A.

Selected cell aliquots were shipped overnight for deformability analysis by ektacytometry to the laboratory of Dr. Kameneva, University of Pittsburgh. Typical images produced by ektacytometry are presented in Figure 9. The light absorption coefficient is relatively high for RBCs containing hemoglobin, which is a red pigment carrying protein, as compared to water, and relatively low for other non-Hb containing cells and the suspending medium. The use of a bandwidth interference filter (380–420 nm) made the Hb containing cells appear as dark objects against the bright background and other non-Hb containing cells. Therefore, one can see a clear evidence of enrichment of Hb containing deformable cells, characteristic for the mature RBCs, in the positive fraction following magnetic separation. These results agree with the findings of the CTV analysis, discussed above, and are further proof of consistency of the CTV method with the already established methods of physical cell analysis.

### Throughput of separation of HSC-derived RBC cultures on MACS columns

The maximum capacity of one LD column recommended by the manufacture is 1.0×10^8^ magnetic cells from a total cell population of 5.0×10^8^ cells. In order to obtain a separation throughput (cell number separated per day) as high as possible, different sample volumes from 1.0×10^8^ to 5.0×10^8^ total cells were tested. It was found that the process of magnetic separation was significantly hampered by column clogging when 5.0×10^8^ total cells was applied to the column. Columns clogged occasionally even for lower number of loaded cells of 2.00×10^8^ total. The clogging was traced back to a large number of platelet-size cells found in batches of HSC-derived RBC cultures, which greatly reduced the column capacity. These platelet- size cells, likely immature platelets and/or released cell nuclei, tend to adhere to each other and are retained in the column, and subsequently are collected in the positive fraction (Figure S5).

The separation results are summarized in File S2 as Table 1. The typical flow rate of cell suspension through the column was about 0.2–0.25 mL/min. The total cell sorting speed per column could be estimated and is also listed. The best cell recovery in the positive fraction was obtained for the feed of 1.00×10^8^ cells, followed by 2 mL buffer rinse. The corresponding cell sorting speed per column was the lowest, however. A relatively high sorting speed and a reproducible cell recovery in the positive fraction were obtained for separation conditions involving 2.50×10^8^ cells in the loaded sample followed by a rinse with 4 mL buffer.

## Discussion

The potential of nearly unlimited amounts of RBCs production from hematopoietic progenitor cell cultures for blood transfusions without the need of donors is appealing [2], [3]. Given the goal of producing RBCs for safe transfusions into humans, not only is it desirable for the final product to contain functioning RBCs but also that it is devoid of contaminating cells (such as nucleated, immature hematopoetic cells). While centrifugation or filtration can remove many such contaminating cells based on differences in the characteristic cell density and size, the presence of paramagnetic (deoxygenated) hemoglobin in the maturing RBC creates an intriguing possibility of magnetically separatiing such cells. In other words, the presence of the deoxygenated hemoglobin in the maturing RBCs provides a natural (intrinsic) magnetic tag offering a unique opportunity for a label-free magnetic separation of maturing erythrocytes from the progenitor cell culture. In this report, we have focused on determining the feasibility of such an approach given the challenges of weak RBC magnetization by the deoxygenated hemoglobin and the large number of cells required for blood transfusion.

In order to better characterize the effect of the intrinsic cell magnetization on separation, we applied the cell magnetophoretic mobility analysis to measure changes in the magnetic cell fractional composition in cultures used for separation, combined with the cell size distributions analysis. The results showed that the hematopoietic progenitor cultures contained cells with sufficient amount of Hb to effect their separation using a commercial, high-gradient magnetic separator (MACS™ system). Interestingly, it was also observed that these putative RBCs and reticulocytes were bigger than the adult donor RBCs. Furthermore, the heterogeneity of the sorted cell product was confirmed by other methods used as a reference standard, namely cell size distribution analysis by Coulter counter, DIC microscopy, and RBC deformability assay. The combination of physical methods probing different aspects of cell physical properties produced a self-consistent picture of the cell culture product as a mixture of cells with the intracellular hemoglobin concentration comparable to that of mature RBCs but being greater in size, more typical of reticulocytes. This type of purely physical analysis, demonstrating that the cells contain nearly normal concentrations of functioning hemoglobin yet are larger than normal, adult RBCs, provides important information about the biophysical characteristics of the hematopoietic progenitor cell culture system, important for further refinement of the method.

The results summarized in File S2 (as Table 1) served the purpose of illustrating the technical challenges of label-free, magnetic RBC sorting using current, commercially available HGMS columns in application to collecting an equivalent of one unit of blood. Assuming 2.5×10^8^ cells sorted per run per column without clogging the column and the availability of an automated process capable of operating 1,000 columns in parallel 24 hours per day (or fewer, larger columns of equivalent total capacity) we estimate that it would take approximately 7 days to produce one unit of blood (2×10^12^ RBCs) from the starting mixture containing 14% maturing RBCs in the HSC culture in a production facility. In the final analysis, the time required for accomplishing the separation demonstrates the limitation of the batch processing of the HSC cultures using magnetic HGMS columns, and challenges associated with scaling up of such a process.

An alternative is a continuous magnetic RBC sorting process, in which the flow-through, magnetic RBC sorter (with the associated deoxygenator) is operated at the volumetric flow rates matching the RBC production rate in the HSC culture. We have demonstrated feasibility of the continuous, flow-through magnetic cell sorting process in the past, albeit in applications to the magnetically labeled cells characterized by much higher magnetophoretic mobility than the deoxygenated RBCs [20]. Nevertheless, the current results provided a rational basis for design of flow-through magnetic sorter for label-free RBC separation, as well as suggested new utilization of the cultured RBCS, such as drug delivery that requires small volumes of the modified RBCs [21]. Thus, a continuous magnetic cell separation system integrated with the in-line deoxygenator could be a promising option for production of the cultured RBCs intended for therapeutic applications.

## Supporting Information

File S1 **Supplemental information for** **Materials and Methods** **section.**
(DOC)

File S2 **Table 1: MACS separation results.**
(DOC)

Figure S1 **The physical properties of the cell change in the course of erythropoiesis.** Cell mean corpuscular hemoglobin concentration (MCHC), magnetophoretic mobility (MM), m, and hydrodynamic diameter are shown at different stages of mammalian erythropoiesis. (A) The mean m value increases with the RBC maturation was calculated from known cell MCHC and diameters found in the literature [12], [13] as described in the text. (B) The broad distribution of the magnetophoretic mobilities at different stages of the RBC maturation determines the mixed composition of the magnetically separated fraction – note significant overlap between RBC and reticulocyte distributions leading to the expected presence of both types of cells in the magnetic fraction (with appreciable admixture of normoblasts).
(TIF)

Figure S2 **Magnetic separation experimental setup under low oxygen conditions.** Photograph of the deoxygenation and magnetic cell separation system and its components. The cells were kept in N2 gas (humidified) atmosphere inside a polyethylene bag compartment in open 50 mL conical tubes rotated on an inclined rotator for 3 hours prior to separation, together with other system components, including media and the magnetic HGMS columns.
(TIF)

Figure S3 **Changes in the partial oxygen pressure, pO2, and oxygen saturation, SO2, of the whole blood with time under experimental conditions shown in Figure S2.**
(TIF)

Figure S4 **Magnetic separation of HSC cultures increases concentration of small cells, by Coulter counter method.** Cell volume distributions of (A) unsorted and (B) sorted cell samples by Coulter counter method. The donor blood RBC size distribution is also shown in (A) for reference. Note enrichment in small cells in the “positive” fraction in (B) indicating enrichment in putative maturing RBCs.
(TIF)

Figure S5 **Evidence of platelet-like cells in the positive fraction of the magnetically separated HSC cultures.**
(TIF)
