# Supplementary material for: Erythrocyte Enrichment in Hematopoietic Progenitor Cell Cultures Based on Magnetic Susceptibility of the Hemoglobin
Source: PLoS One. 2012 Aug 27;7(8):e39491. doi: 10.1371/journal.pone.0039491 (PMC3428333; doi:10.1371/journal.pone.0039491)
Supplement: File S1 — Supplemental information for Materials and Methods section. (DOC) [file pone.0039491.s001.doc]

**Supporting Information**

Erythrocyte enrichment in hematopoietic progenitor cell cultures based on magnetic susceptibility of the hemoglobin

**Xiaoxia Jin^a,d^, Stewart Abbot^b^, Xiaokui Zhang^b^, Lin Kang^b^, Vanessa Voskinarian-Berse^b^, Rui Zhao^c^, Marina V. Kameneva^c^, Lee R. Moore^a^, Jeffrey J. Chalmers^d^, Maciej Zborowski^a^**

^a^Department of Biomedical Engineering, Cleveland Clinic, 9500 Euclid Ave., Cleveland, OH 44195

^b^Celgene Cellular Therapeutics, 7 Powderhorn Drive, Warren, NJ 07059

^c^McGowan Institute for Regenerative Medicine, University of Pittsburgh, 450 Technology Drive, Pittsburgh, PA 15219;

^d^William G. Lowrie Department of Chemical and Biomolecular Engineering, The Ohio State University, 140 W. 19^th^ Ave., Columbus, OH 43210

Introduction

In this study, we have tested if the label-free magnetic separation of red blood cells (RBCs) could be adapted for enrichment of maturing RBCs from large-scale hematopoietic cell cultures (see Main Text). We have used a variety of physical methods to determine the properties of the maturing RBCs, including quantitative measurement of the distribution of RBC magnetization in cell mixtures by analytical cell magnetophoresis (using Cell Tracking Velocimetry, CTV). The label-free magnetic separation method is based on changes in the cell size and its magnetic susceptibility related to the increasing concentration of intracellular hemoglobin. During differentiation, erythroid cells progressively become smaller and increase their hemoglobin content, exhibit chromatin condensation, and become enucleated, which contribute to their increasing magnetophoretic mobility (Supporting Information Figure S1).

Red blood cell magnetic susceptibility

The observation that blood may exhibit measurable magnetic susceptibility is attributed to Michael Faraday (in 1800s) and was followed by physical measurements (on hemoglobin isolates) and quantum mechanical model of heme electron spin contribution to molar susceptibility of hemoglobin by Linus Pauling and coworkers [1]. Those early studies and a later observation of the interference of venous blood with the nuclear magnetic resonance imaging spurred interest in the quantitative determination of the effect of blood oxygenation on its magnetic susceptibility. According to previous studies [2,3,4,5,6,7,8] the RBC volume magnetic susceptibility is the weighted sum of the susceptibilities of its components:

$\chi_{RBC}=\varphi_{H_{2}O}\chi_{H_{2}O}+(1-S)\varphi_{Hb}\chi_{deoxyHb}+\varphi_{Hb}\chi_{globin}$ (1)

where *φ_H2O_*, *φ_Hb_* are the volume fractions of water and hemoglobin in the RBC, respectively, *χ_H2O_* (= -0.719 × 10^-6^, cgs, or -9.04 × 10^-6^, SI), *χ_deoxyHb_*, and *χ_globin_* are the volume magnetic susceptibilities of water, the four ferroheme groups in deoxy Hb, and globin, respectively. *S* is the oxygen saturation of oxyhemoglobin, *S* = 1 for a fully oxygenated RBC (oxy RBC), *S* = 0 for a fully deoxygenated RBC (deoxy RBC).

The volume fraction occupied by water in a RBC is determined from the volume fraction of the hemoglobin,

$\varphi_{H_{2}O}=1-\varphi_{Hb}=1-V_{m,Hb}c_{Hb}=0.735$ (2)

where *V_m,Hb_* = 48.227 L/mol is the molar volume of hemoglobin, and *c_Hb_* = 5.5 × 10^-3^ mol/L (corresponding to 350 g/L with a molecular weight *M_w,Hb_* = 64,450 g/mol) is the intracellular hemoglobin concentration in one mature RBC [7,9].

The volume magnetic susceptibility of metHb RBC has a similar form as the one above but contributed mainly by metHb:

$\chi_{RBC}=\varphi_{H_{2}O}\chi_{H_{2}O}+Z\varphi_{Hb}\chi_{metHb}+\varphi_{Hb}\chi_{globin}$ (3)

where *Z* is the fraction of Hb converted to metHb, and *χ_metHb_* is the magnetic susceptibility of methemoglobin.

The numerical values of the remaining terms in Supporting Eqns (1) and (3) are as follows: *φ_Hb_χ_deoxyHb_* = *c_Hb_χ_m,deoxyHb_*, where *χ_m,deoxyHb_ =* 50,893 × 10^-9^ L/mol is the molar susceptibility of deoxy Hb, *φ_Hb_χ_globin_* = *c_Hb_χ_m,globin_*, where *χ_m,globin_ =* -37,832 × 10^-9^ L/mol [3] is the molar susceptibility of the globin, and *φ_Hb_χ_metHb_* = *c_Hb_χ_m,metHb_*, where *χ_m,metHb_ =* 56,000 × 10^-9^ L/mol is the molar susceptibility of met Hb [10,11]. By inserting these terms into Supporting Eqns (1) and (3), one obtains the volume magnetic susceptibilities for RBCs in cgs unit system, which could be converted to SI unit system by multiplication of 4π:

$\chi_{oxyRBC}=-7.36\times10^{-7}(cgs)\text{ or }-9.25\times10^{-6}(SI)$, fully oxy RBC, *S* = 1 (4)

$\chi_{deoxyRBC}=-4.56\times10^{-7}(cgs)\text{ or }-5.74\times10^{-6}(SI)$, fully deoxy RBC, *S* = 0 (5)

$\chi_{metHbRBC}=-4.28\times10^{-7}(cgs)\text{ or }-5.38\times10^{-6}(SI)$, fully metHb RBC, Z = 1 (6) $\chi_{H2O}=-7.19\times10^{-7} \text{(}cgs\text{)} \text{or} -9.04\times10^{-6} \text{(}SI\text{)}$, water (7)

Note that diamagnetic contributions to the RBC magnetic susceptibility predominate, even for deoxy Hb (Supporting Eqn (5)) and metHb (Supporting Eqn (6)) RBCs, and that only the magnetic susceptibility of the oxyHb RBC (Supporting Eqn (4)) is lower than that of water (Supporting Eqn (7)), albeit by a very small fraction.

Reversible RBC magnetization by deoxygenation of HSC-derived RBC cultures

A Glove-Bag™ inflatable glove chamber (Cole Parmer, Vernon Hills, IL), filled with nitrogen (Medipure™ nitrogen, concentration > 99%, Praxair, Inc., Danbury, CT) was used to deoxygenate HSC- derived RBC cultures (Supporting Information Figure S2). The nitrogen gas was humidified by bubbling through water. After passing through a 0.2 µm filter, the humidified gas entered into the glove bag through a tubing coupler, which was inserted into the gas port built into the glove bag. The actual gas flow rate into the glove bag was not measured, but the gas pressure was controlled by a N_2_ gas cylinder pressure regulator at about 50 kPa.

The efficiency of the deoxygenation system to removing O_2_ from blood was first tested on 5 mL WB placed in an inclined 50 mL conical tube affixed to a stirring rotator in N_2_ atmosphere. The relatively large surface area of the 50 mL conical tube and its rotating motion facilitated a rapid gas exchange in a small sample volume smeared over the internal surface of the conical tube. About 0.1 mL samples were taken at different time points for blood oximetry. Partial pressure of oxygen (pO_2_) and oxygen saturation (sO_2_) were measured by a blood gas analyzer (ABL715, Radiometer, Denmark). An optimal exposure to N_2_ time (3 hrs.) for achieving the lowest blood pO_2_ (below 3 mmHg) corresponding to the lowest blood oxygen saturation, *S* (below 4%) was selected (Figure S3) and then applied to sorting of the cultured RBC samples.

Cell size distribution

An automated cell counter, Z2™ Coulter Counter® (Beckman Coulter Inc., Fullerton, CA, USA), with a 70 μm aperture, a sample volume of 0.5 mL and a diameter range setting between 4 and 14 μm was used to measure cell concentration and size distribution in the magnetically sorted fractions (Figure S4).

RBC deformability

Typical images produced by ektacytometry are presented in Figure S5. The light absorption coefficient is relatively high for RBCs containing hemoglobin, which is a red pigment carrying protein, **as compared to water, and relatively low for other non-Hb containing cells and the suspending medium. The use of a bandwidth interference filter (380 - 420 nm) made the Hb containing cells appear as dark objects against the bright background and other non-Hb containing cells. The cluster of small, round, bright objects is interpreted as evidence of presence of platelets in the HSC culture (as indicated in Figure S5).**

Supporting Information References

1. Pauling L, Coryell CD (1936) The magnetic properties and structure of hemoglobin, oxyhemoglobin and carbonmonoxyhemoglobin. Proceedings of the National Academy of Science 22: 210-216.

2. Cerdonio M, Congiucastellano A, Calabrese L, Morante S, Pispisa B, et al. (1978) Room-Temperature Magnetic-Properties of Oxyhemoglobin and Carbonmonoxy-Hemoglobin. Proceedings of the National Academy of Sciences of the United States of America 75: 4916-4919.

3. Cerdonio M, Morante S, Torresani D, Vitale S, DeYoung A, et al. (1985) Reexamination of the evidence for paramagnetism in oxy- and carbonmonoxyhemoglobins. Proc Natl Acad Sci U S A 82: 102-103.

4. Fabry ME, George RCS (1983) Effect of Magnetic-Susceptibility on Nuclear Magnetic-Resonance Signals Arising from Red-Cells - a Warning. Biochemistry 22: 4119-4125.

5. Plyavin YA, Blum EY (1983) Magnetic parameters of blood cells and high gradient paramagnetic and diamagnetic phoresis. Magnetohydrodynamics 19: 349-359.

6. Savicki JP, Lang G, Ikedasaito M (1984) Magnetic-Susceptibility of Oxyhemoglobins and Carbonmonoxyhemoglobins. Proceedings of the National Academy of Sciences of the United States of America-Biological Sciences 81: 5417-5419.

7. Spees WM, Yablonskiy DA, Oswood MC, Ackerman JJH (2001) Water proton MR properties of human blood at 1.5 Tesla: Magnetic susceptibility, T-1, T-2, T-2* and non-Lorentzian signal behavior. Magnetic Resonance in Medicine 45: 533-542.

8. Weisskoff RM, Kiihne S (1992) Mri Susceptometry - Image-Based Measurement of Absolute Susceptibility of Mr Contrast Agents and Human Blood. Magnetic Resonance in Medicine 24: 375-383.

9. Lichtman M, Beutler E, Kaushansky K, Kipps T, Seligsohn U, et al. (2005) Williams Hematology: McGraw-Hill Professional.

10. Hackett S, Hamzah J, Davis TM, St Pierre TG (2009) Magnetic susceptibility of iron in malaria-infected red blood cells. Biochim Biophys Acta 1792: 93-99.

11. Pauling L, Coryell CD (1936) The Magnetic Properties and Structure of the Hemochromogens and Related Substances. Proc Natl Acad Sci U S A 22: 159-163.

12. Marieb EN, Hoehn K (2007) Human Anatomy and Physiology: Benjamin-Cummings Publishing Co.

13. Wojda U, Noel P, Miller JL (2002) Fetal and adult hemoglobin production during adult erythropoiesis: coordinate expression correlates with cell proliferation. Blood 99: 3005-3013.
