# Supplementary material for: Erythrocyte Enrichment in Hematopoietic Progenitor Cell Cultures Based on Magnetic Susceptibility of the Hemoglobin
Source: PLoS One. 2012 Aug 27;7(8):e39491. doi: 10.1371/journal.pone.0039491 (PMC3428333; doi:10.1371/journal.pone.0039491)
Supplement: File S2 — Table 1: MACS separation results. (DOC) [file pone.0039491.s002.doc]

Table 1 MACS separation results

| Cell # and volume per column | Rinse  (mL) | Positive cell #  Mean ± SD | $\frac{\text{Positive cell }\#\text{ }}{\text{feed cell \#}}$(%) | Overall recovery  Mean ± SD (%) | Number of tubes* | Number of columns** | Sorting speed (s^-1^) |
| --- | --- | --- | --- | --- | --- | --- | --- |
| 1.0×10^8^ / 1.0 mL | 2 | (1.57 ± 0.12)×10^7^ | 15.7 ± 1.2 | 93.9 ± 4.8 | 3 | 14 | 1.1×10^5^ |
| 1.5×10^8^ / 1.5 mL | 3 | (1.86 ± 0.25)×10^7^ | 12.4 ± 1.7 | 98.6 ± 4.7 | 3 | 3 | 1.2×10^5^ |
| 2.0×10^8^ / 2.0 mL | 4 | (2.13 ± 0.46)×10^7^ | 10.7 ± 2.3 | 98.3 ± 3.8 | 4 | 16 (3) | 1.2×10^5^ |
| 2.5×10^8^ / 2.5 mL | 4 | (3.44 ± 0.31)×10^7^ | 13.8 ± 1.2 | 96.4 ± 3.2 | 1 | 6 (1) | 1.4×10^5^ |
| 2.5×10^8^ / 2.5 mL | 5 | (1.94 ± 0.13)×10^7^ | 7.8 ± 0.5 | 101.5 ± 6.5 | 2 | 7 | 1.2×10^5^ |
| 3.0×10^8^ / 3.0 mL | 5 | (2.37 ± 0.26)×10^7^ | 7.9 ± 0.9 | 98.8 ± 3.5 | 2 | 6 (2) | 1.4×10^5^ |

* Number of tubes used for the HSC culture collection (listed here for internal reference). **Total number of columns used for magnetic RBC separation at a condition listed in Column #1 (thus the total number of cells processed for that condition is a product of entries listed in Columns #1 and #7). Numbers in parentheses refer to the number of clogged columns resulting in a failed experiment. “Sorting speed” was calculated as the total number of cells loaded onto the MACS column divided by the time it took to complete the separation.
