# Supplementary figures and images for: Erythrocyte Enrichment in Hematopoietic Progenitor Cell Cultures Based on Magnetic Susceptibility of the Hemoglobin
Source: PLoS One. 2012 Aug 27;7(8):e39491. doi: 10.1371/journal.pone.0039491 (PMC3428333; doi:10.1371/journal.pone.0039491)

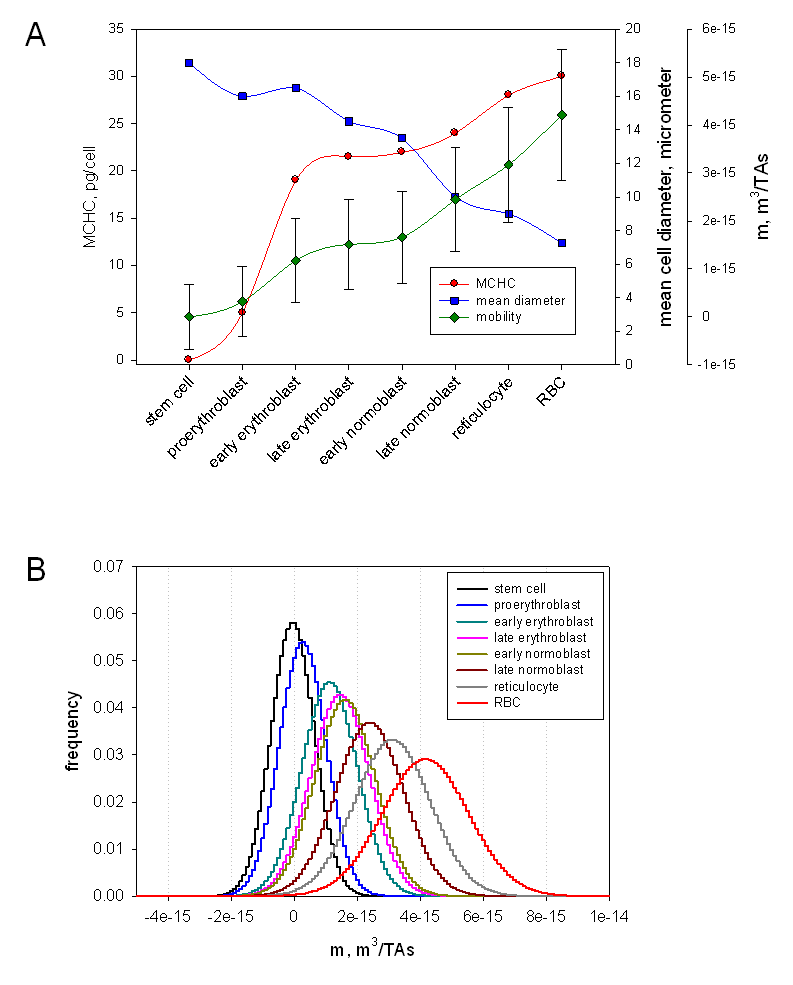

Supplement: Figure S1 — The physical properties of the cell change in the course of erythropoiesis. Cell mean corpuscular hemoglobin concentration (MCHC), magnetophoretic mobility (MM), m, and hydrodynamic diameter are shown at different stages of mammalian erythropoiesis. (A) The mean m value increases with the RBC maturation was calculated from known cell MCHC and diameters found in the literature [12], [13] as described in the text. (B) The broad distribution of the magnetophoretic mobilities at different stages of the RBC maturation determines the mixed composition of the magnetically separated fraction – note significant overlap between RBC and reticulocyte distributions leading to the expected presence of both types of cells in the magnetic fraction (with appreciable admixture of normoblasts). (TIF) [file pone.0039491.s003.tif]

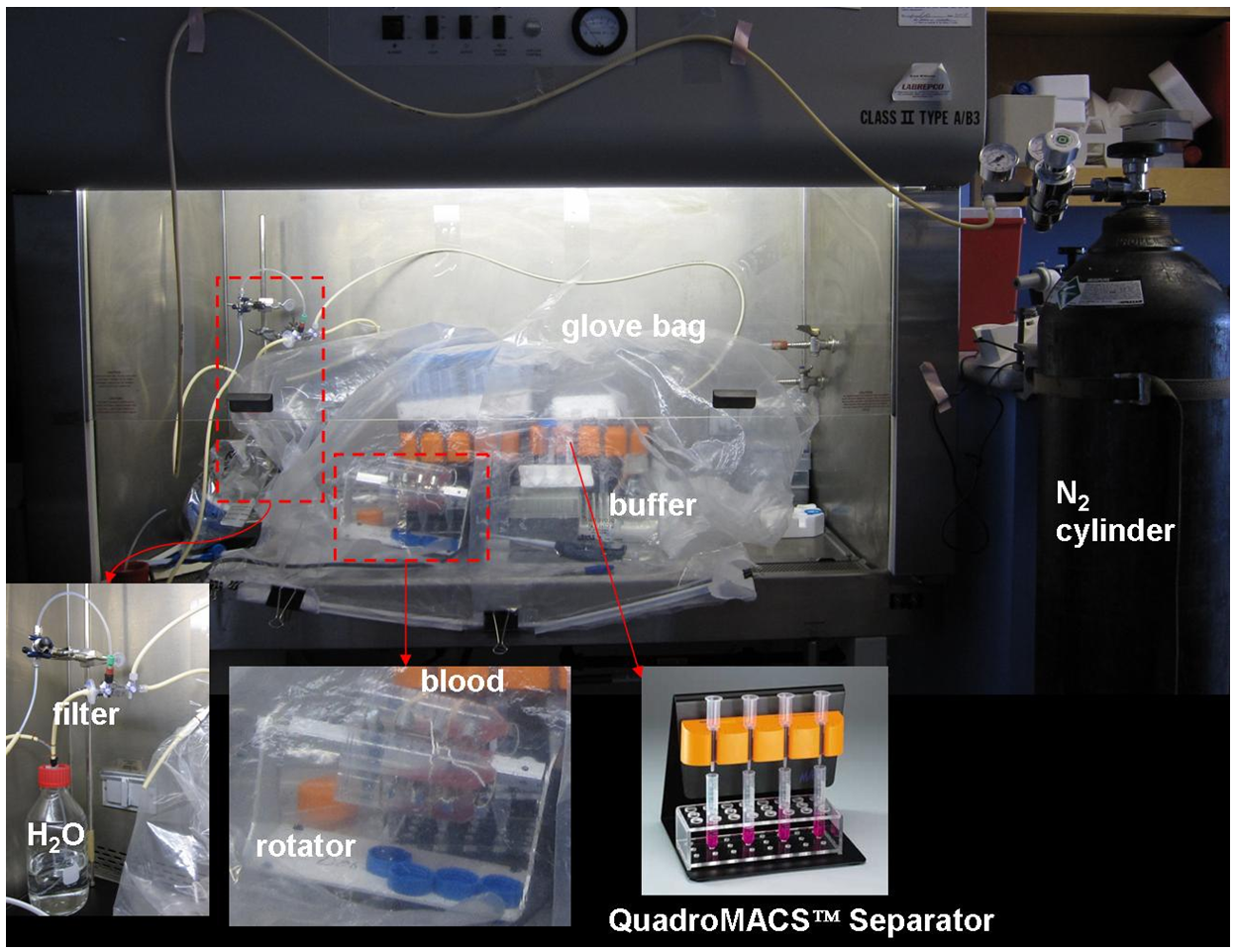

Supplement: Figure S2 — Magnetic separation experimental setup under low oxygen conditions. Photograph of the deoxygenation and magnetic cell separation system and its components. The cells were kept in N2 gas (humidified) atmosphere inside a polyethylene bag compartment in open 50 mL conical tubes rotated on an inclined rotator for 3 hours prior to separation, together with other system components, including media and the magnetic HGMS columns. (TIF) [file pone.0039491.s004.tif]

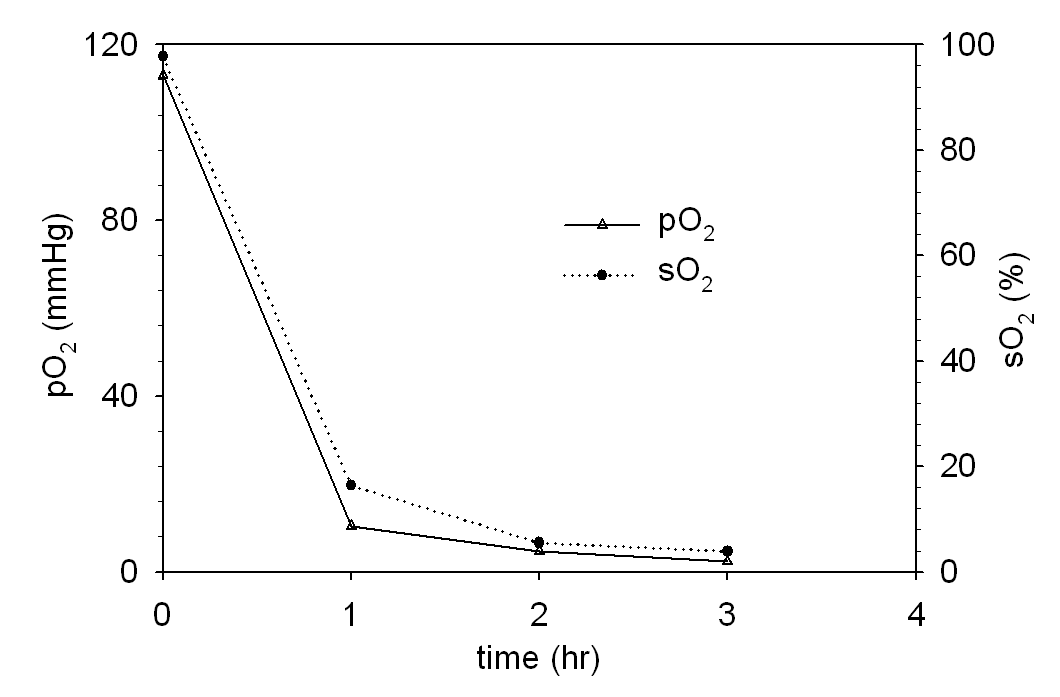

Supplement: Figure S3 — Changes in the partial oxygen pressure, pO2, and oxygen saturation, SO2, of the whole blood with time under experimental conditions shown in Figure S2. (TIF) [file pone.0039491.s005.tif]

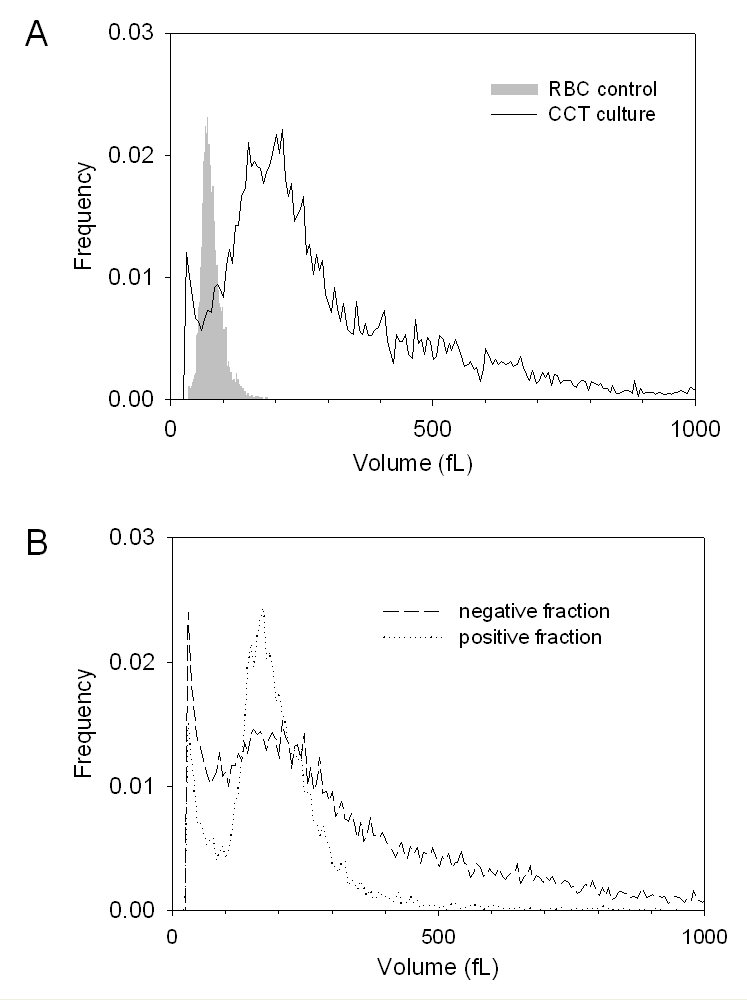

Supplement: Figure S4 — Magnetic separation of HSC cultures increases concentration of small cells, by Coulter counter method. Cell volume distributions of (A) unsorted and (B) sorted cell samples by Coulter counter method. The donor blood RBC size distribution is also shown in (A) for reference. Note enrichment in small cells in the “positive” fraction in (B) indicating enrichment in putative maturing RBCs. (TIF) [file pone.0039491.s006.tif]

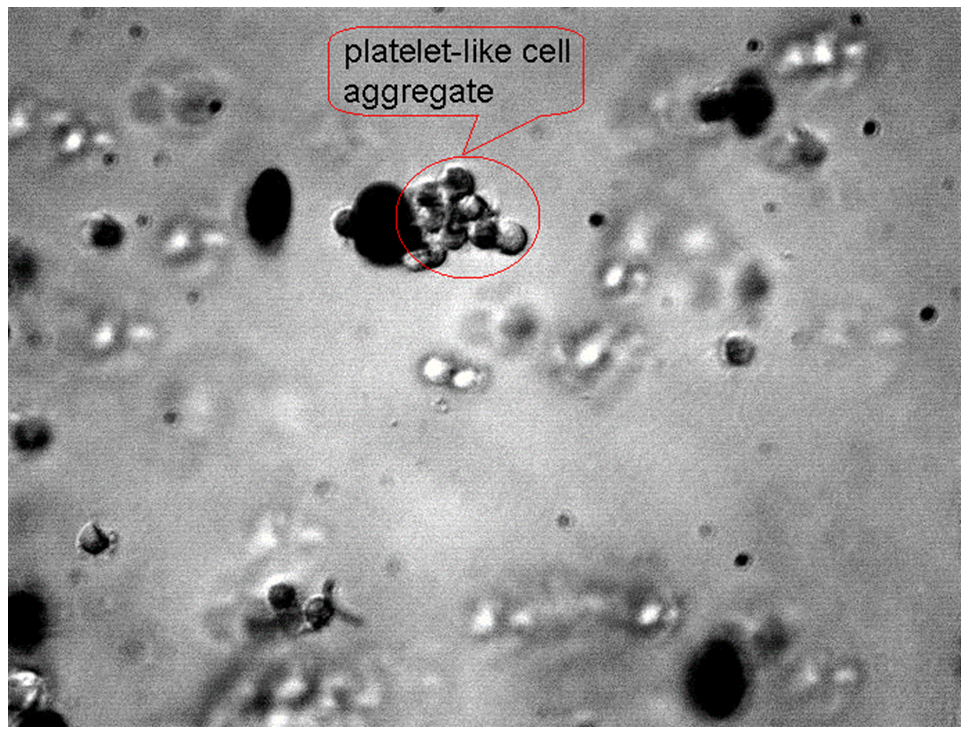

Supplement: Figure S5 — Evidence of platelet-like cells in the positive fraction of the magnetically separated HSC cultures. (TIF) [file pone.0039491.s007.tif]
